# Resident-led organizational initiatives to reduce burnout and improve wellness

**DOI:** 10.1186/s12909-019-1756-y

**Published:** 2019-11-27

**Authors:** Sundus Mari, Rachel Meyen, Bo Kim

**Affiliations:** 1 Department of Psychiatry, Child and Adolescent Psychiatry Fellowship, Boston Children’s Hospital, Boston, MA USA; 2 Department of Psychiatry, Harvard Medical School, Boston, MA USA; 3 Department of Psychiatry, Partners Geriatric Psychiatry Fellowship, Massachusetts General Hospital, Boston, MA USA; 4 Center for Healthcare Organization and Implementation Research, VA Boston Healthcare System, Boston, MA USA

**Keywords:** Burnout, Wellness, Quality improvement, Physician trainees

## Abstract

**Background:**

Professional burnout among medical trainees has been identified as a national concern in need of attention. A significant challenge for residency programs is designing and implementing effective strategies to promote resident wellness and reduce burnout. Emerging evidence highlights the importance of developing organizational changes targeting physician burnout.

**Methods:**

To address this critical need, Harvard South Shore (HSS) Psychiatry Residency Training Program aimed to assess burnout among residents, identify areas for wellness-related growth, and implement strategies for organizational change to reduce burnout and increase wellness. We aligned closely to the Standards for Quality Improvement Reporting Excellence (SQUIRE) 2.0 guidelines to systematically approach planning, conducting, and evaluating this quality improvement effort. We developed a wellness action team and assessed burnout using the Copenhagen Burnout Inventory (CBI). We also conducted a survey to investigate high opportunity areas for wellness-related growth and using this data we designed and implemented four organizational initiatives to (i) improve residents’ on-call experience, (ii) increase social activities, (iii) support preventative care, and (iv) promote wellness education. We then re-assessed burnout 1 year after implementation and performed two-sample t-tests to compare CBI scores. We additionally gathered and analyzed feedback from residents on the implemented organizational initiatives’ relevance to wellness and their well-being.

**Results:**

There was an overall clinically meaningful reduction in burnout averaged among all residents that participated. Participants indicated that fitness-oriented activities were most likely to lead to change in wellness habits.

**Conclusion:**

Our implemented wellness program was resident-led and involved continuous feedback from both residents and leadership. Given that there may be multiple factors that affect resident burnout, future studies involving a control group could help reveal whether our intervention contributed to the change in burnout scores we observed.

**Electronic supplementary material:**

The online version of this article (10.1186/s12909-019-1756-y) contains supplementary material, which is available to authorized users.

## Background

Burnout has been defined as a syndrome of emotional exhaustion, depersonalization or cynicism, and feelings of inadequacy regarding one’s work with patients [1]. Professional burnout affects at least fifty-percent of US physicians [2] with medical trainees at particularly high risk [3]. Due to the inherent challenges of medical training, residents may feel exhausted, disenchanted, detached, or cynical about their work. Suicidal ideations among physicians have been related to their degree of burnout and suicide rates are higher among US physicians than the general population [4, 5]. In addition, burnout may lead to physician attrition and higher rates of depression, substance use and relationship difficulties [6, 7]. Burnout has also been shown to negatively impact quality of patient care [8–10]. Resident physicians struggling with burnout are more likely to perform medical errors [11] and their patients may have an increased risk of mortality [12]. Along the same vein, there is some evidence that physician wellness such as self-reported good health and positive personal health practices are associated with increased patient satisfaction and improved patient adherence to treatment [13].

In response to these outcomes, in 2017, the Accreditation Council for Graduate Medical Education (ACGME) revised their Common Program Requirements to address the need to reduce burnout and improve wellness among residents. Residency Programs have taken different approaches to meet this requirement but there is insufficient evidence for their efficacy [14]. Studies on the effects of work-hour limitations have shown variable outcomes [15–17] but Bolster et al. demonstrated that decreasing duty hours alone did not improve resident wellness and may even have had negative impacts on resident education [18]. Specific wellness programming is often developed to focus on individual residents by encouraging their participation in wellness activities. For instance, Runyan et al’s work used a wellness curriculum to encourage residents to incorporate self-reflection skills, journaling, gratitude and mindful breathing into their daily schedules [19]. Others introduced self-care workshops, communication and stress management trainings or process groups [20–22]. However, in isolation, these strategies miss the opportunity of developing system-wide changes to decrease burnout and improve wellness. Although the literature does identify the need for organizational structures to promote wellness, the evidence for specific, concrete strategies remains limited.

The Mayo Clinic Program on Physician Well-being [8] has been a leader in the field of developing organizational leadership strategies to reduce physician burnout. They seek to identify problems and develop solutions that can be practically implemented within a large health care organization. The strategies seek to empower individual members to identify problems and develop inexpensive and feasible solutions. They have been found to be effective within a large healthcare association, among three clinically diverse sites (Minnesota, Arizona, and Florida) and the implementation of these strategies is correlated with measurable improvements in burnout [8].

Based on the Mayo Clinic’s strategies, our residency program has built their own wellness program to assess burnout, identify areas for wellness-related growth, and implement strategies for organizational change. In this project we aim to assess burnout among residents at Harvard South Shore (HSS) and to evaluate the effectiveness of our own wellness program by gathering residents’ perspectives.

## Methods

### Setting and participants

Harvard South Shore is an ACGME-accredited Psychiatry Residency Training Program based within the VA Boston Healthcare System and affiliated with Harvard Medical School. The program lasts four years and has an average of 30 residents with about 8 residents per post-graduate year (PGY). The residents rotate through multiple different sites within the VA Boston Healthcare System, Harvard-affiliated hospitals and community hospitals.

Current organizational dynamics at HSS allow for residents to drive program change through the development of resident-led action teams, and the Program Evaluation Committee (PEC). This committee allows for residents to provide feedback and be an integral part of program policy changes made by leadership. In addition, residents are encouraged to participate in quality improvement projects to learn about systems-level issues and to enhance their leadership and communication skills. Prior to this project, rates of burnout among current HSS residents and target areas for wellness-related growth had not been formally assessed. As part of ACGME’s efforts to emphasize wellness in residency programs, HSS leadership recognized the possibility of burnout among residents and encouraged the development of a resident-led wellness action team.

Four residents of different postgraduate levels formed the Wellness Action Team in March 2017 and initiated a quality improvement project based on Mayo Clinic’s strategies. We closely aligned phases of our project to the Standards for Quality Improvement Reporting Excellence (SQUIRE 2.0) guidelines to systematically approach planning, conducting and evaluating this quality improvement effort (www.squire-statement.org). This project has been reviewed according to VA Boston Healthcare System (Boston, Massachusetts, USA) procedures and has been determined to be a quality improvement activity that is classified as non-research, requiring no further oversight by the health system’s research committees.

Our project ran from June 2017 to June 2018 and all residents present during that time were invited to participate (Total of 39 residents: Class 2017 and Class 2018 with 7 residents each, Class 2019 and Class 2020 with 8 residents each and Class 2021 with 9 residents).

### Description of the wellness program

We administered a survey to identify the residency program’s current strengths and weaknesses in promoting wellness (Additional file 2). The etiology of resident burnout is multifactorial, and varies among specialties, programs, and even among post-graduate years within a program. We therefore chose to directly survey residents to determine the improvement efforts that would be most relevant to HSS. This strategy also helped avoid the possible impact of a generation gap on the wellness programming which has been identified as a possible limitation of wellness initiatives [23, 24].

**Fig. 1:**
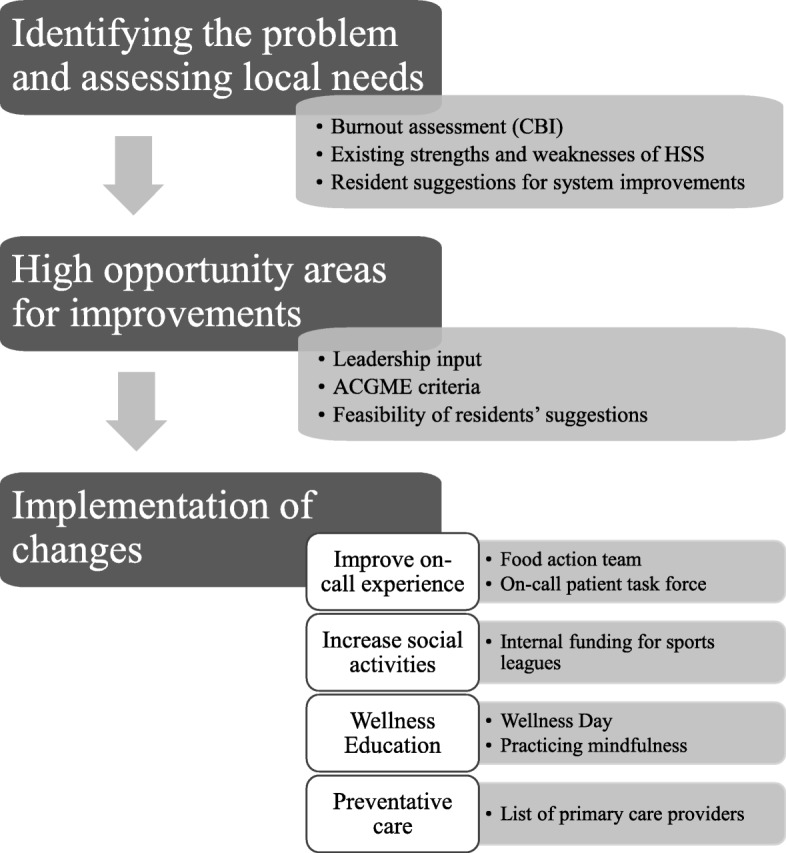
Summary of wellness changes implemented as a result of residents’ and leadership suggestions. Abbreviations: CBI indicates Copenhagen Burnout Inventory; HSS, Harvard South Shore Psychiatry Residency Program; ACGME, Accreditation Council for Graduate Medical Education

Building on the survey findings, together with HSS leadership and in consideration of feasibility of residents’ suggestions, we decided which improvement efforts to pursue. Almost 50% of the residents listed their on-call experience as the main factor contributing to burnout. We therefore formed both an ‘on-call patient task force’ and a ‘food action team’ to improve work flow and to provide healthier food options to residents while they are on call. The ‘on-call patient task force’ consists of an interdisciplinary group including residents, nurses, staff physicians and hospital administrators. They performed an investigation akin to a root-cause analysis to identify aspects of the admission process that may benefit from improvement. Increasing social activities outside of work was the second most common request by residents. This was addressed by collaborating with HSS program leadership to allocate internal funding to promote resident participation in sports leagues (e.g. soccer and flag football) with the added benefit of increasing exercise; another important aspect of wellness. These sports leagues met for weekly games after regular residency working hours. To meet ACGME criteria, leadership emphasized the importance of preventative care as well as promoting wellness through education. This led to the addition of a designated ‘wellness day’ to our didactic curriculum and inclusion of mindfulness activities on regular didactic days (weekly). We also provided residents with a list of primary care providers (e.g. psychotherapists, nutritionists, dentists and primary care physicians) to facilitate access to preventative care. A summary of these changes is depicted in Fig. 1.

After leadership approved the addition of a wellness day as a way of promoting wellness in the program, members of the wellness action team started planning different activities for the day. The American Medical Association [25] defines six key aspects of wellness: nutrition, fitness, emotional health, preventative care, financial health, and mindset and behavior adaptability (understanding/navigating how to thrive in your work environment) [25]. Based on AMA’s definition, we developed a list of various activities that would cover these aspects of wellness. We then actively solicited suggestions and feedback from all residents at monthly meetings to develop the different sessions for wellness day. It is worth noting that residents unanimously voted against including a session on financial health to allow for more time to be spent on other activities. To educate residents about nutrition, we brought in a nutritionist to perform a ‘healthy eating’ cooking demonstration. A Tai Chi class in addition to a dedicated hour of gym (or outdoor) time enabled us to address the fitness aspect. For emotional health we offered an art therapy class and a gratitude exercise and for mindset and behavior adaptability we included a session on physician mental health and mind-body medicine. To address the preventative care aspect of wellness, we included a fatigue-mitigation exercise. We asked residents to come prepared to share their own strategies to avoid fatigue and then compiled this information on a poster board. This was later hung in the on-call rooms to serve as a reminder of the various approaches.

### Measures

We considered different burnout assessment inventories and opted to use the Copenhagen Burnout Inventory (CBI); a well validated psychometric test available in the public domain [26]. The CBI is a 19-item survey divided into three scales: personal burnout, which measures the degree of both physical and psychological exhaustion experienced by the person; work-related burnout, which looks at what proportion of their exhaustion they perceive to be related to their work; and client-related burnout, which measures how much their work with clients (in our case patients) relates to their fatigue (Additional file 1). Looking at these different domains separately puts emphasis on what causes burnout rather than the symptoms experienced by subjects. The score of each scale ranges from 0 to 100 with higher values indicating increased probability of burnout. While some studies have used cut-off scores (e.g. 50 or higher) to indicate burnout, these have not been validated [27–29]. We therefore opted to use them as continuous variables assessing degree of burnout and focusing on trends over time rather than numerical scores. The developers of the scale have also suggested that a difference of 5 points or more for one survey respondent may be considered clinically significant [26].

Feedback from residents was gathered using paper-based surveys handed out during resident meetings (Additional files 3 and 4). Participation in the surveys and all other aspects of the project was entirely voluntary. Surveys did not collect any identifying information beyond post-graduate year and all discussions of survey results were carried out using aggregate data.

### Wellness program evaluation

We administered the CBI in June 2017 which represents the last month of a post-graduate year and the PGY-4 or Class of 2017 graduating the program. At the end of the academic year, in June 2018, we re-administered the CBI to assess whether there has been an overall reduction in burnout among residents. We held Wellness Day in February 2018 and at the end of the day we administered a survey to assess perceived utility of Wellness Day. In addition, we gathered feedback from residents regarding possible wellness day improvements (Additional file 3) and we attempted to identify which initiatives of the wellness program (including wellness day) were perceived to be most relevant to residents’ sense of wellness (Additional file 4).

### Analysis

Feedback regarding to what extent residents felt that Wellness Day addressed AMA’s key aspects of wellness was converted to percentages of residents who responded with ‘very’ or ‘extremely’ on the survey. Similarly, information gathered regarding the perceived effectiveness of Wellness Day and the other interventions was graphed in this manner. We did not link data collected at the beginning and end of the project to protect anonymity, and we performed two-sample unequal variances t-tests to compare mean CBI sub-scores between classes and pre-and post-implementation of the project.

## Results

**Fig. 2:**
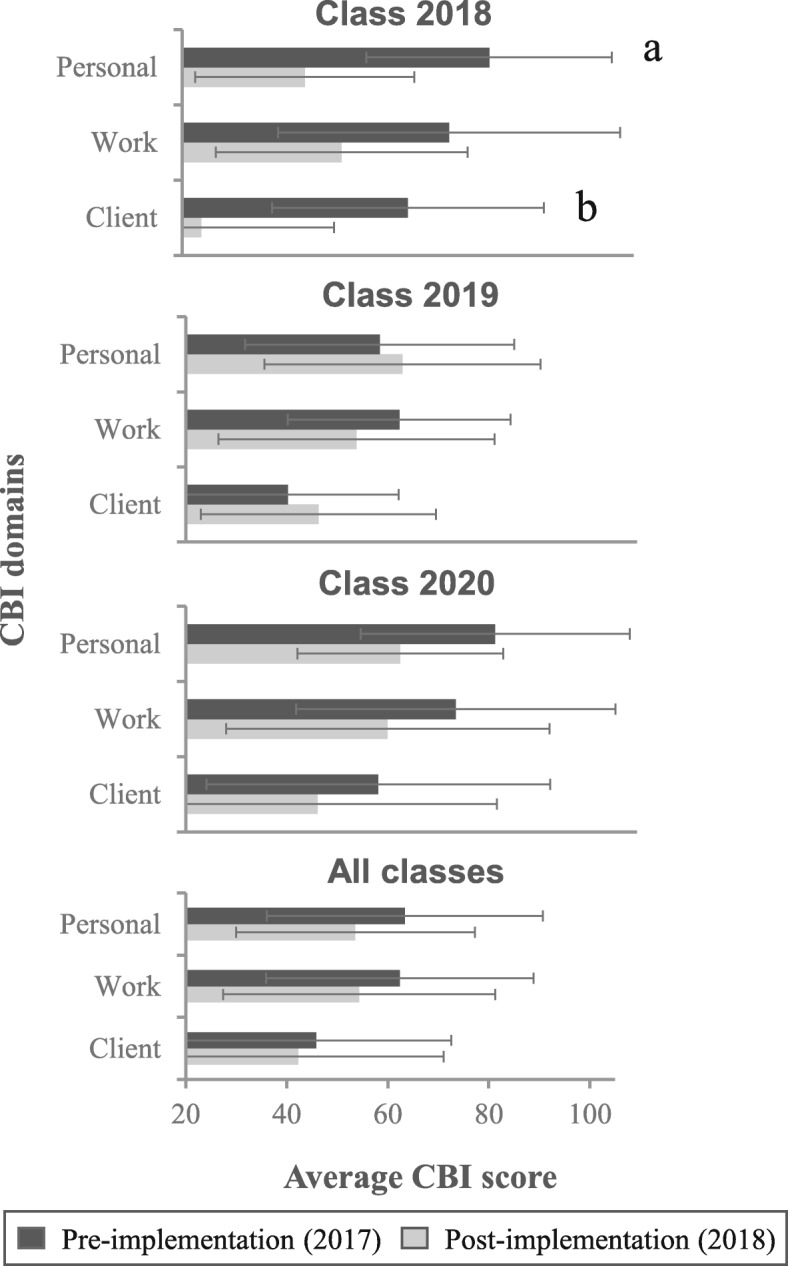
Average Copenhagen Burnout Inventory (CBI) scores in each domain (personal, work, client) pre-and post-implementation of wellness initiative at Harvard South Shore Psychiatry Residency Program, 2017–2018. Class 2020: 4 out of 8 residents responded to the survey pre-and post-implementation showing a reduction in burnout in each domain, progressing from post-graduate year (PGY) 1 to PGY-2. Class 2019: 7 out of 8 residents responded pre-implementation and 5 post-implementation showing an increase in burnout in the personal and client domain and a reduction in the work domain. They progressed from PGY-2 to PGY-3. Class 2018: 3 out of 7 residents responded pre-and post-implementation showing a reduction in burnout in each domain while progressing from PGY-3 to PGY-4. a: t = 2.35, *p* = 0.050. b: t = 2.98, *p* < 0.05. All residents: 14 out of 23 residents responded pre-implementation and 12 post-implementation showing a reduction in burnout in each domain

While a total of 39 residents in the program were present, we only included data from residents that could participate across the entire span of the one-year project (i.e. we excluded the graduating class in 2017 (7 residents) and the incoming class in 2018 (Class 2021 with 9 residents). 14 out of 23 residents (Total of 61%: 4 out of 8 from Class 2020, 7 out of 8 from Class 2019 and 3 out of 7 from Class 2018) responded to the surveys at the start of the project and 12 (Total of 52%: 4 out of 8 from Class 2020, 5 out of 8 from Class 2019 and 3 out of 7 from Class of 2018) after the intervention, one year later. The average CBI scores of the individual classes pre-and post-intervention are shown in Fig. 2 (see Table 1 for means and standard deviations charted in Fig. 2).

**Table 1:** Average CBI scores pre-and post-intervention

	Class 2018		Class 2019		Class 2020		All classes	
	Pre (*n* = 3)	Post (*n* = 3)	Pre (*n* = 7)	Post (*n* = 5)	Pre (*n* = 4)	Post (*n* = 4)	Pre (*n* = 14)	Post (*n* = 12)
Personal	78 (+/−26)	43 (+/−21)	57 (+/− 25)	61 (+/− 26)	78 (+/− 25)	60 (+/− 19)	63 (+/− 27)	54 (+/− 24)
Work	70 (+/− 32)	50 (+/− 24)	60 (+/− 21)	52 (+/− 26)	71 (+/− 30)	58 (+/− 30)	62 (+/− 26)	54 (+/− 27)
Client	63 (+/− 23)	24 (+/− 25)	39 (+/− 21)	45 (+/− 22)	56 (+/− 32)	45 (+/− 34)	46 (+/− 27)	42 (+/− 29)

Average Copenhagen Burnout Inventory (CBI) scores in each domain (personal, work, client) pre-and post-implementation of wellness initiative at Harvard South Shore Psychiatry Residency Program, 2017–2018. Numbers of participants from each class are shown and standard deviations of the mean scores are indicated between brackets

The differences in scores between classes pre-intervention were not statistically significant. However, post-intervention there was a significant difference in the client domain score between Class of 2019 and Class of 2018 (t = 2.78, *p* = 0.02). Overall, there appears to have been a clinically meaningful (i.e. difference of 5 points or more, based on the scale’s developer’s suggestion [26]) downward trend in burnout in all three domains but only the reductions in CBI scores in the client domain of Class of 2018 reached statistical significance (t = 2.98, *p* < 0.05). Class of 2019 started out with the lowest burnout scores and was the only class that demonstrated an upward trend.

**Fig. 3:**
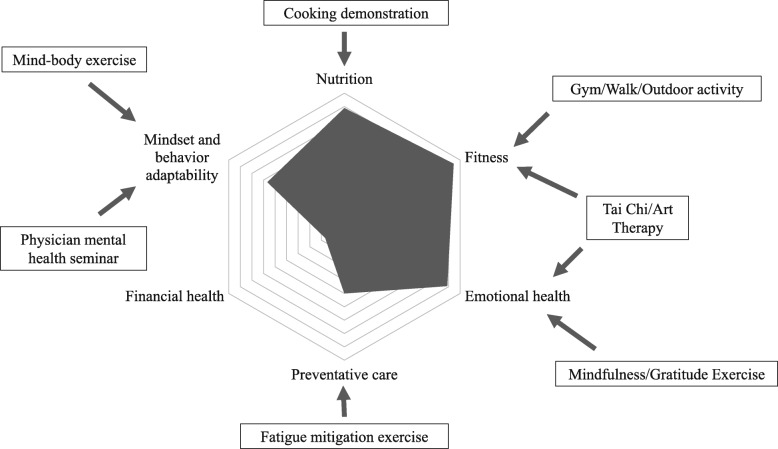
Radial map showing the percentage of survey respondents (*n* = 18) who responded with ‘very’ or ‘extremely’ when asked to what extent the different Wellness Day sessions addressed the American Medical Association’s key aspects of wellness (nutrition, fitness, emotional health, preventative care, financial health, mindset and behavior adaptability). The corresponding Wellness Day sessions are outlined around the map

**Fig. 4:**
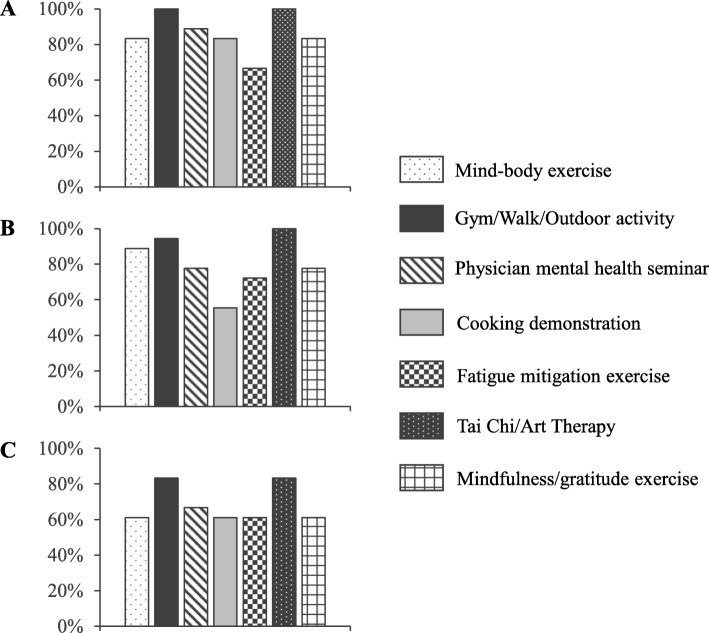
Percentage of survey respondents (n = 18) who responded with ‘very’ or ‘extremely’ to post-wellness day survey questions addressing (**a**) Perceived relevance of sessions to sense of wellness, (**b**) Perceived effectiveness of sessions in enhancing understanding of wellness, and (**c**) Perceived extent to which sessions will change wellness habits

The majority of residents (94%) felt that Wellness Day addressed AMA’s fitness aspect the most, followed by the nutrition and emotional health (89%) aspects (Fig. 3). As residents had voted not to include a session on financial health, it is not surprising that only 17% (3 residents) felt that this was addressed by Wellness Day. In terms of the different activities, all residents perceived the gym/walk/outdoor activity and the Tai Chi/Art Therapy activity as most relevant to their sense of wellness (Fig. 4, Chart A). Only 67% of residents thought that the fatigue mitigation exercise was relevant to their sense of wellness. A similar trend was observed when asked about the effectiveness of the different sessions in promoting understanding of wellness (Fig. 4, Chart B). 100% of residents thought that the Tai Chi/Art Therapy session was most effective, followed by 94% for the gym/walk/outdoor activity and 89% for the mind-body exercise. Here, only 55% of residents perceived the cooking demonstration as effective. With regards to whether the different sessions will have an impact on residents’ wellness habits (Fig. 4, Chart C), again most residents felt that the gym/walk/outdoor activity was most impactful (83%) followed by an even distribution (about 61%) for all other activities.

In addition to assessing the impact of Wellness Day, we also attempted to identify what aspects of the overall project residents found most helpful in improving wellness. Most residents (85%) felt that the overall changes addressed AMA’s nutrition aspect the most, followed by fitness (62%) and emotional health (54%). Other than the switch in nutrition and fitness, the distribution is similar to Fig. 3, with only 7% of residents feeling that the financial health aspect was addressed.

**Fig. 5:**
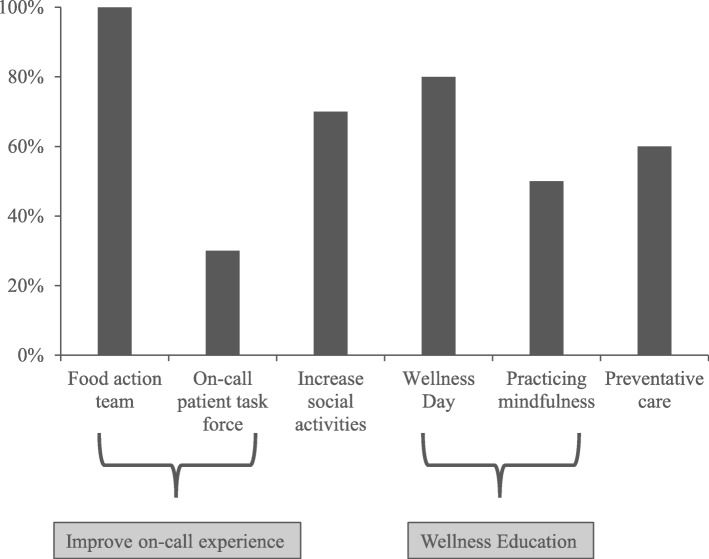
Percentage of survey respondents (*n* = 10) who responded with ‘very’ or ‘extremely’ when asked how relevant the implemented changes were to their sense of wellness. Food action team and on-call patient task force correspond to improving the on-call experience (see Fig. 1) and Wellness Day and practicing mindfulness represent wellness education. Increase social activities was achieved through internal funding allocated to sponsoring sports leagues for residents. Preventative care corresponds to providing residents with a list of preventive care providers

As shown in Fig. 5, all residents felt that the food action team was most relevant to their sense of wellness. 80% thought that Wellness Day was most relevant, followed by 70% for the increase in social activities. Only 30% perceived the on-call patient task force as relevant to their sense of wellness.

## Discussion

Our efforts at HSS to reduce burnout and improve wellness among psychiatry residents involved four organizational initiatives – (i) improve residents’ on-call experience, (ii) increase social activities, (iii) support preventative care, and (iv) promote wellness education. Measured using the CBI, all postgraduate years showed an overall clinically meaningful reduction in burnout following the initiatives’ implementation.

The average baseline burnout scores were slightly higher than those observed in other studies of residents, but they showed a similar pattern with higher scores in the personal and work domain and lower client-related burnout scores [29–31]. As we did not gather any additional demographic data from participants to help ensure anonymity, it is difficult to predict why our cohort appears to have a higher level of burnout than others. Nonetheless, it’s an important finding that provides useful information for program leadership and emphasizes the need for wellness initiatives.

While we were able to demonstrate a reduction in burnout, the extent of change differs between classes, with Class of 2019 showing an upward trend in burnout for the personal and client domains. As mentioned earlier, the etiology of burnout is multifactorial and residents’ responsibilities change as they progress in post-graduate years. For instance, generally the intern year tends to be quite demanding across different residency programs and even specialties, due to the steep learning curve one encounters after graduating medical school and realizing one’s responsibility for patients. This has been shown in other studies as an increase in burnout scores at the end of the academic year, particularly in the client domain [29, 30]. It is therefore possible that finishing their intern year was a large contributor to the reduction in burnout in Class of 2020. Likewise, as residents start their fourth and final year of residency, they may have gained confidence in their clinical skills making them less prone to burnout (Class 2018). In addition, at HSS PGY-4 s have no further call responsibilities and more than half of the residents listed their on-call experience as the main factor contributing to burnout. After the intern year, PGY-3 is arguably the most demanding year at HSS. There is a large call burden (in terms of total hours spent on call) in addition to a significant increase in outpatient responsibilities. This may explain the increase in burnout in the personal and client domain for Class 2019. Interestingly, their average burnout score in the work domain had a small drop again indicating the potential effect of other factors on burnout beyond the wellness program. Continuous assessment of burnout across the entirety of the program (four years) may help identify in further detail some of the confounding factors that contribute to a change in burnout from one year to the next.

Beyond the measurement of burnout using the CBI, Wellness Day and the various improvements were also well received by residents. We attempted to address the residents’ request to improve the on-call experience by forming a food action team as well as an on-call patient task force. The food action team successfully improved the overall quality and quantity of food available when residents are on call. All residents perceived that this improvement was most relevant to their sense of wellness. On the contrary, only 30% thought that the on-call patient task force was relevant. This task force was implemented with the intention to improve work-flow and reduce workload on the individual resident when they are on call. However, unlike the mostly simple objective of the food action team, the task force needs to enact system-level changes that are likely more intricate and gradual. This underscores the need for projects such as ours to address resident burnout and wellness through organizational changes. On an individual level, residents will likely only feel the impact of this initiative over time.

Due to the residents’ enthusiasm for Wellness Day and the reduction seen in overall burnout scores (while fully recognizing that numerous other factors may have affected the scores, as discussed above), program leadership agreed to incorporate an annual Wellness Day into the didactic curriculum. All other aspects of the project (e.g. weekly mindfulness, on-call patient task force, weekly sports leagues) are ongoing after completion of this pilot quality improvement project. Overall residents perceived the fitness activities (gym, walk, outdoor activity, Tai Chi) as well as Art Therapy most relevant to their sense of wellness, most effective in enhancing their understanding of wellness and most likely to encourage change in their own wellness habits. While all these opportunities continue to be available on regular working days, we did not assess whether residents actually incorporated them into their daily work schedule. Future research could address this and use the information for planning further wellness initiatives. This also highlights the importance of gathering resident feedback prior to developing wellness programs. While residents had voted against a session on financial health, many suggested to include this in the post-wellness day survey (Additional file 3). The Wellness Action Team will continue as a Wellness Committee in the program evaluation committee. This will ensure that there is continuous assessment and evaluation of the residency program with regards to burnout and wellness and further changes can be made as appropriate.

### Limitations

While we found reduction in overall burnout, due to the lack of a control group and previously mentioned confounding factors, these changes can’t be attributed to our wellness program. Studies have shown that other factors such as the working environment, personality traits, conflicts at home and relationship difficulties have a significant impact on burnout. A study in Taiwan demonstrated that only 5.4% of the variance in CBI scores could be explained by residents’ coping strategies [32]. For future expansions of the project, a control group might help in teasing out the effects of the wellness program on burnout scores. Moreover, although our findings are comparable with those observed in other programs, our sample size was small and limited to psychiatry residents at one institution and hence may not be representative of other resident cohorts.

Anonymity enabled residents to provide survey responses without risk of identification, but prevented us from identifying residents at risk for other issues such as insomnia and clinical depression, that are often co-morbid with significant burnout [6, 7]. Future projects should ensure that residents at risk are directed to the existing employee assistance program without compromising confidentiality. In addition, similarly to other programs [33], HSS should consider assessing sleep, depression and anxiety as individual factors that may affect trainee’s ability to provide the best possible patient care. Prior research has shown that physician wellness is associated with increased patient satisfaction [13]. Since the ultimate goal of quality improvement projects is to improve patient care, future research could also assess effects of burnout on patient care and outcomes.

## Conclusions

After the introduction of ACGME’s new wellness requirements, residency programs have been struggling to design and implement effective strategies to reduce resident burnout and improve wellness. Although programs have taken different approaches, many have not been program-wide, and the evidence for the effectiveness of these initiatives remains limited. Our project was resident-led and involved continuous feedback from both residents and leadership. This allowed the interventions to fill existing gaps rather than a one size fits all application. However, future research involving a control group is necessary to establish whether our wellness program had an effect on burnout scores. In addition, expansion of the project to include other resident cohorts might help in identifying and evaluating strategies to address burnout that can be adapted to other programs. It could also be used to develop a wellness curriculum which has been suggested by other programs [19, 34]. Importantly, these future efforts should carefully examine the extent to which effective wellness enhancement approaches are common or heterogenous across international residency settings, in order to accurately understand the relevance of our wellness program for a broader, international scientific community.

## Additional files

Additional file 1: Copenhagen Burnout Inventory; Adapted burnout measurement instrument/survey. (DOCX 23 kb)

Additional file 2: System improvements for residents’ wellness; Survey to identify the residency program’s current strengths and weaknesses in promoting wellness. (DOCX 21 kb)

Additional file 3: February Wellness Day Survey; Survey to gather feedback from residents regarding possible wellness day improvements. (DOCX 23 kb)

Additional file 4: Outcome of Wellness Initiatives; Survey to identify which initiatives (including wellness day) were perceived to be most relevant to residents’ sense of wellness. (DOCX 19 kb)

## Data Availability

The datasets used and analyzed during the current study are available from the corresponding author on reasonable request.

## Abbreviations

ACGME: Accreditation Council for Graduate Medical Education
AMA: American Medical Association
CBI: Copenhagen Burnout Inventory
HSS: Harvard South Shore
PEC: Program Evaluation Committee
PGY: Post-graduate Year
SQUIRE: Standards for Quality Improvement Reporting Excellence
US: United States
VA: Veterans’ Administration
