# Supplementary material for: Resident-led organizational initiatives to reduce burnout and improve wellness
Source: BMC Med Educ. 2019 Nov 27;19:437. doi: 10.1186/s12909-019-1756-y (PMC6880512; doi:10.1186/s12909-019-1756-y)
Supplement: Supplementary file 1 — Copenhagen Burnout Inventory; Adapted burnout measurement instrument/survey. (DOCX 23 kb) [file 12909_2019_1756_MOESM1_ESM.docx]

Copenhagen Burnout Inventory (Adapted from Copenhagen Burnout Inventory^1^)

Please indicate your PGY-level:

- PGY-1
- PGY-2
- PGY-3
- PGY-4

**Personal burnout**

1. How often do you feel tired?

- Always
- Often
- Sometimes
- Seldom
- Never/Almost never

2. How often are you physically exhausted?

- Always
- Often
- Sometimes
- Seldom
- Never/Almost never

3. How often are you emotionally exhausted?

- Always
- Often
- Sometimes
- Seldom
- Never/Almost never

4. How often do you think: ”I can’t take it anymore”?

- Always
- Often
- Sometimes
- Seldom
- Never/Almost never

5. How often do you feel worn out?

- Always
- Often
- Sometimes
- Seldom
- Never/Almost never
- 6. How often do you feel weak and susceptible to illness?
- Always
- Often
- Sometimes
- Seldom
- Never/Almost never

**Work-related burnout**

7. Is your work emotionally exhausting?

- To a very high degree
- To a high degree
- Somewhat
- To a low degree
- To a very low degree

8. Do you feel burnt out because of your work?

- To a very high degree
- To a high degree
- Somewhat
- To a low degree
- To a very low degree

9. Does your work frustrate you?

- To a very high degree
- To a high degree
- Somewhat
- To a low degree
- To a very low degree

10. Do you feel worn out at the end of the working day?

- Always
- Often
- Sometimes
- Seldom
- Never/Almost never

11. Are you exhausted in the morning at the thought of another day at work?

- Always
- Often
- Sometimes
- Seldom
- Never/Almost never

12. Do you feel that every working hour is tiring for you?

- Always
- Often
- Sometimes
- Seldom
- Never/Almost never

13. Do you have enough energy for family and friends during leisure time?

- Always
- Often
- Sometimes
- Seldom
- Never/Almost never

**Client-related burnout**

14. Do you find it hard to work with clients?

- To a very high degree
- To a high degree
- Somewhat
- To a low degree
- To a very low degree

15. Do you find it frustrating to work with clients?

- To a very high degree
- To a high degree
- Somewhat
- To a low degree
- To a very low degree

16. Does it drain your energy to work with clients?

- To a very high degree
- To a high degree
- Somewhat
- To a low degree
- To a very low degree

17. Do you feel that you give more than you get back when you work with clients?

- To a very high degree
- To a high degree
- Somewhat
- To a low degree
- To a very low degree

18. Are you tired of working with clients?

- Always
- Often
- Sometimes
- Seldom
- Never/Almost never

19. Do you sometimes wonder how long you will be

able to continue working with clients?

- Always
- Often
- Sometimes
- Seldom
- Never/Almost never

Reference:

1. Kristensen TS, Borritz M, Villadsen E, Christensen KB. The Copenhagen Burnout Inventory: A new tool for the assessment of burnout. *Work & Stress.* 2005;19(3):192-207.
