# Supplementary material for: Resident-led organizational initiatives to reduce burnout and improve wellness
Source: BMC Med Educ. 2019 Nov 27;19:437. doi: 10.1186/s12909-019-1756-y (PMC6880512; doi:10.1186/s12909-019-1756-y)
Supplement: Supplementary file 2 — System improvements for residents’ wellness; Survey to identify the residency program’s current strengths and weaknesses in promoting wellness. (DOCX 21 kb) [file 12909_2019_1756_MOESM2_ESM.docx]

System improvements for residents’ wellness (Administered through survey monkey: [www.surveymonkey.com](http://www.surveymonkey.com))

The purpose of this survey is to identify areas of system improvements for residents’ wellness at HSS. All answers are anonymous and will be collected in aggregate form. Thank you for your participation.

1. Please indicate your PGY-level:

- PGY-1
- PGY-2
- PGY-3
- PGY-4

1. What does wellness mean to you? (Free text answer response format)

|  |
| --- |

1. What are HSS’s strengths in fostering wellness? Please explain. (Free text answer response format)

|  |
| --- |

1. What are “high opportunity areas” for HSS to target improved wellness? (i.e. what areas should we focus our attention to when working to improve wellness?) Please explain. (Free text answer response format)

|  |
| --- |

1. AMA defines 6 key aspects of wellness: nutrition, fitness, emotional health, preventative care, financial health, and mindset and behavior adaptability (understanding/navigating how to thrive in your work environment). On a scale of 1-10 (10 being the highest level of wellness), please rate your personal wellness in the following categories:
   1. Nutrition 0-10
   2. Fitness 0-10
   3. Emotional health 0-10
   4. Preventative care 0-10
   5. Financial health 0-10
   6. Mindset and behavior adaptability 0-10
2. Do you have any concrete suggestions or ideas for promoting resident wellness at HSS? (Free text answer response format)

|  |
| --- |

1. How often should the HSS community talk formally about wellness together? (Free text answer response format)

|  |
| --- |

1. In what setting(s) should HSS address resident wellness? Choose all that apply.
   1. Continue with current format: “ombuds” once/month
   2. “All residents” meetings with leadership
   3. Anonymous wellness surveys
   4. Individual check-ins
   5. Mass e-mails
   6. Focus groups for each PGY level
   7. Other (please specify) (Free text answer response format)

|  |
| --- |

1. With what people at HSS would you like to discuss resident wellness? Choose all that apply.
   1. Program Director and Associate Program Director
   2. Chief residents
   3. Urgent care attendings/leadership
   4. Inpatient attendings
   5. Inpatient director
   6. Other (please specify) (Free text answer response format)

|  |
| --- |
