# Supplementary material for: Resident-led organizational initiatives to reduce burnout and improve wellness
Source: BMC Med Educ. 2019 Nov 27;19:437. doi: 10.1186/s12909-019-1756-y (PMC6880512; doi:10.1186/s12909-019-1756-y)
Supplement: Supplementary file 3 — February Wellness Day Survey; Survey to gather feedback from residents regarding possible wellness day improvements. (DOCX 23 kb) [file 12909_2019_1756_MOESM3_ESM.docx]

February Wellness Day Survey

Thank you for participating at our inaugural Wellness Day. We are interested in learning about your experience so that we can make improvements for future wellness days.

Please indicate your PGY-level:

- PGY-1
- PGY-2
- PGY-3
- PGY-4

1. AMA defines 6 key aspects of wellness: nutrition, fitness, emotional health, preventative care, financial health, and mindset and behavior adaptability (understanding/navigating how to thrive in your work environment). To which extent did Wellness Day address each of the following aspects?

| CATEGORY | Not very | Somewhat | Very | Extremely |
| --- | --- | --- | --- | --- |
| Nutrition |  |  |  |  |
| Fitness |  |  |  |  |
| Emotional health |  |  |  |  |
| Preventative care |  |  |  |  |
| Financial health |  |  |  |  |
| Mindset and behavior adaptability |  |  |  |  |

1. How relevant were the following topics to your sense of wellness?

|  | Not very | Somewhat | Very | Extremely | Could not participate |
| --- | --- | --- | --- | --- | --- |
| Resident-led mind-body medicine exercise |  |  |  |  |  |
| Gym/Walk/Outdoor activity |  |  |  |  |  |
| Physician mental health seminar |  |  |  |  |  |
| Cooking demonstration by nutritionist |  |  |  |  |  |
| Resident-led fatigue mitigation exercise |  |  |  |  |  |
| Tai Chi/Art Therapy |  |  |  |  |  |
| Resident-led mindfulness/gratitude exercise |  |  |  |  |  |

1. How effective were the following sessions in enhancing your understanding of wellness?

|  | Not very | Somewhat | Very | Extremely | Could not participate |
| --- | --- | --- | --- | --- | --- |
| Resident-led mind-body medicine exercise |  |  |  |  |  |
| Gym/Walk/Outdoor activity |  |  |  |  |  |
| Physician mental health seminar |  |  |  |  |  |
| Cooking demonstration by nutritionist |  |  |  |  |  |
| Resident-led fatigue mitigation exercise |  |  |  |  |  |
| Tai Chi/Art Therapy |  |  |  |  |  |
| Resident-led mindfulness/gratitude exercise |  |  |  |  |  |

1. To what extent do you feel that the sessions will change your wellness habits?

|  | Not very | Somewhat | Very | Extremely | Could not participate |
| --- | --- | --- | --- | --- | --- |
| Resident-led mind-body medicine exercise |  |  |  |  |  |
| Gym/Walk/Outdoor activity |  |  |  |  |  |
| Physician mental health seminar |  |  |  |  |  |
| Cooking demonstration by nutritionist |  |  |  |  |  |
| Resident-led fatigue mitigation exercise |  |  |  |  |  |
| Tai Chi/Art Therapy |  |  |  |  |  |
| Resident-led mindfulness/gratitude exercise |  |  |  |  |  |

1. How would you improve wellness day for next year? Please consider the 6 key aspects of wellness as laid out in Question 1. (Free text answer response format)

|  |
| --- |
