# Supplementary material for: Resident-led organizational initiatives to reduce burnout and improve wellness
Source: BMC Med Educ. 2019 Nov 27;19:437. doi: 10.1186/s12909-019-1756-y (PMC6880512; doi:10.1186/s12909-019-1756-y)
Supplement: Supplementary file 4 — Outcome of Wellness Initiatives; Survey to identify which initiatives (including wellness day) were perceived to be most relevant to residents’ sense of wellness. (DOCX 19 kb) [file 12909_2019_1756_MOESM4_ESM.docx]

Outcome of Wellness Initiatives

Based on your recommendations earlier this year, all of you and the wellness action team have attempted to address the following issues:

- Changing the culture in Urgent Care: Food action team, On-call patient task force
- Social activities outside of work: Flag Football, Soccer
- Promoting education/wellness through didactics, talks, seminars: February Wellness Day, Stretching and Mindfulness during ‘all residents’ meetings
- Preventative care: List of psychotherapists, primary care physicians, nutritionists and dentists

We are interested in learning about your experience with these changes so that we can make improvements for next year.

Please indicate your PGY-level:

- PGY-1
- PGY-2
- PGY-3
- PGY-4

1. AMA defines 6 key aspects of wellness: nutrition, fitness, emotional health, preventative care, financial health, and mindset and behavior adaptability (understanding/navigating how to thrive in your work environment). To which extent did the changes made this year address each of the following aspects?

| CATEGORY | Not very | Somewhat | Very | Extremely |
| --- | --- | --- | --- | --- |
| Nutrition |  |  |  |  |
| Fitness |  |  |  |  |
| Emotional health |  |  |  |  |
| Preventative care |  |  |  |  |
| Financial health |  |  |  |  |
| Mindset and behavior adaptability |  |  |  |  |

1. How relevant were the following changes to your sense of wellness?

|  | Not very | Somewhat | Very | Extremely | Could not participate |
| --- | --- | --- | --- | --- | --- |
| Food action team |  |  |  |  |  |
| On-call patient task force |  |  |  |  |  |
| Flag football/Soccer |  |  |  |  |  |
| February Wellness Day |  |  |  |  |  |
| Stretching/Mindfulness during ‘all residents’ meetings |  |  |  |  |  |
| List of primary care providers |  |  |  |  |  |

1. Do you have other concrete suggestions or ideas for promoting resident wellness at HSS? Please consider the 6 key aspects of wellness as laid out in Question 1. (Free text answer response format)

|  |
| --- |
